# Supplementary material for: Intra-prostatic tumour evolution, steps in metastatic spread and histogenomic associations revealed by integration of multi-region whole-genome sequencing with histopathological features
Source: Genome Med. 2024 Feb 19;16:35. doi: 10.1186/s13073-024-01302-x (PMC10877771; doi:10.1186/s13073-024-01302-x)
Supplement: Supplementary file 1 — Additional file 1. Supplementary figures supporting findings described in the main text. [file 13073_2024_1302_MOESM1_ESM.docx]

1. **Pairwise plots of SNV CCFs**


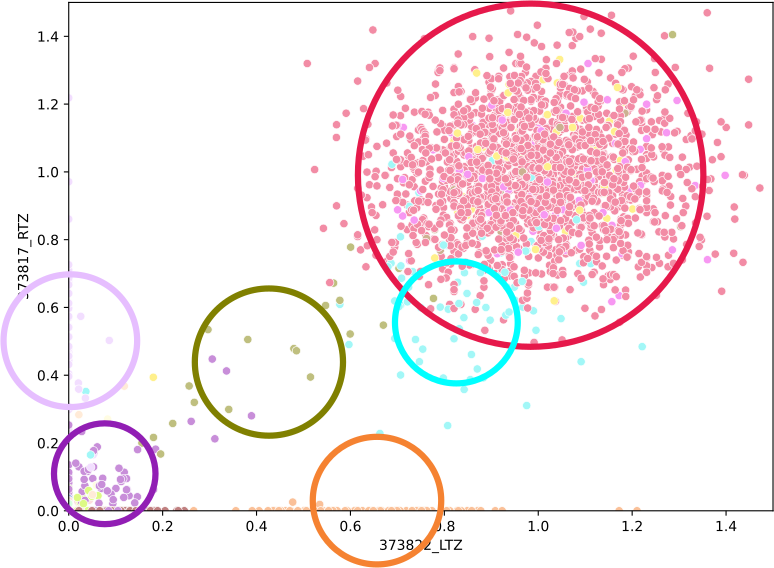


Fig. S1. Pairwise plots of SNV CCFs were used to visually confirm accurate calling of clusters. The median CCF value of each cluster was taken as the CCF of the subclone. The CCF of the truncal cluster must be 1.

2. **Patient #02**

Copy number profile


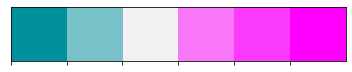


Fig. S2. Copy number profile plots are colour-coded as above, with dark and light blue representing homozygous (0) and heterozygous (1) deletions respectively, grey denoting a diploid copy number (2), and shades of pink representing increasing total copy number counts (>= 3).


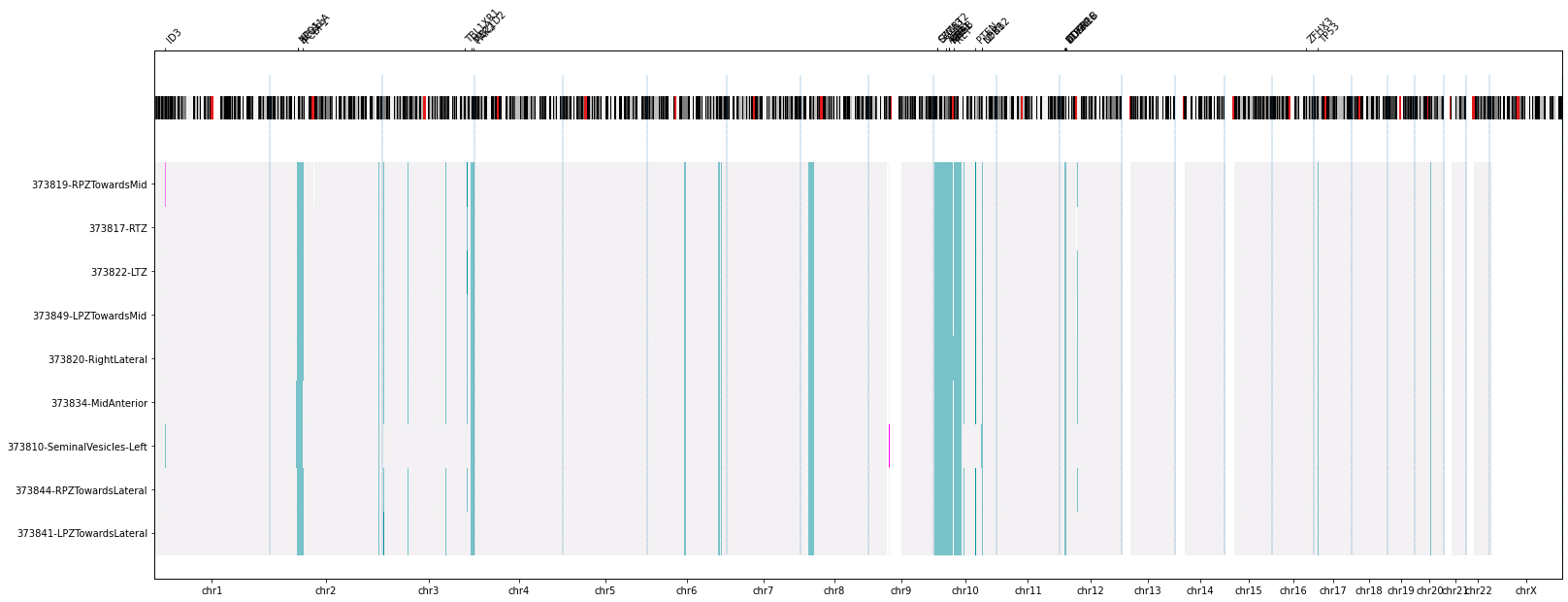
Fig. S3. Copy number profile for patient #02, with genomic coordinates along the X axis and sample names along the Y axis (bottom). Cytoband data for each chromosome (to help with visualisation of centromeres which are depicted as red stripes) and known driver genes present in the CNAs are annotated at the top of the plot. Lymph node samples 5A, 6A and 7A were assumed to have the same profiles as the majority of the intra-prostatic samples; hence the copy number profiles for these samples are not shown.

CCF-cluster plot


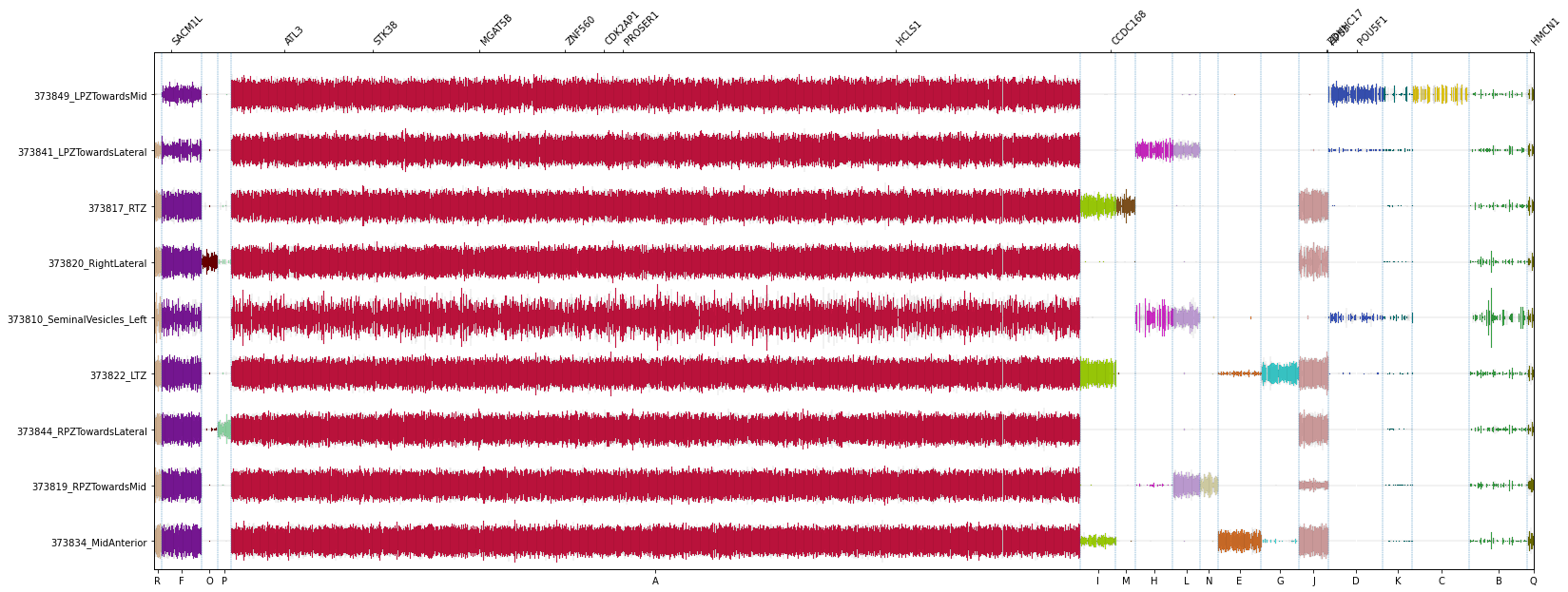
Fig. S4. In the CCF-cluster plot, SNVs represented as vertical lines are grouped by their cluster annotation along the X axis (bottom) and sample names are along the Y axis. The height of each SNV is proportional to its copy number adjusted CCF. Missense mutations in known coding regions are annotated along the top of the X axis. Low tumour purity samples (e.g. 373810_SeminalVesicles_Left) are more noisy. SNVs from clusters K, B, and Q cannot be placed on the phylogenetic tree and may represent noise. Hence these SNVs were ignored.

3. **Patient #08**

Copy number profile


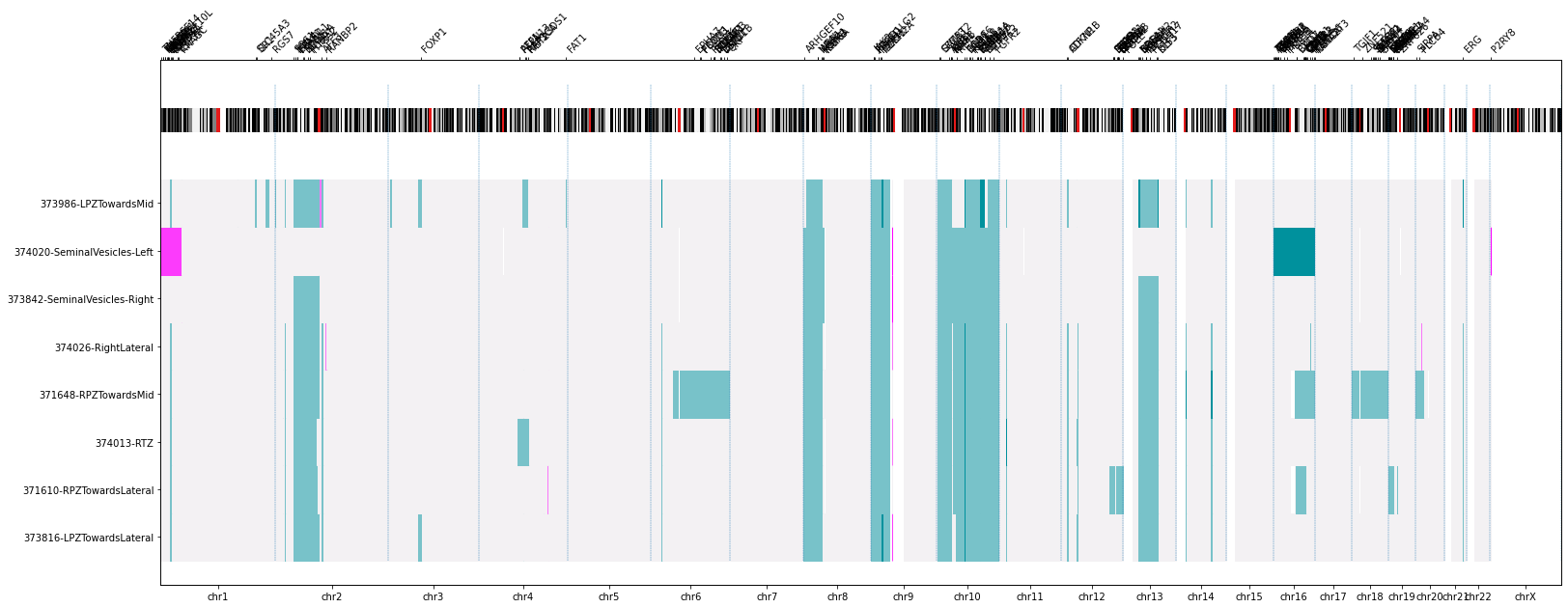
Fig. S5. 374020-SeminalVesicles_Left is of lower tumour purity (0.06) and some CNA calls (e.g. 1p amplification, 16 deletion, and missed CNA calls 2p LOH, 13q LOH) are inaccurate.

CCF-cluster plot


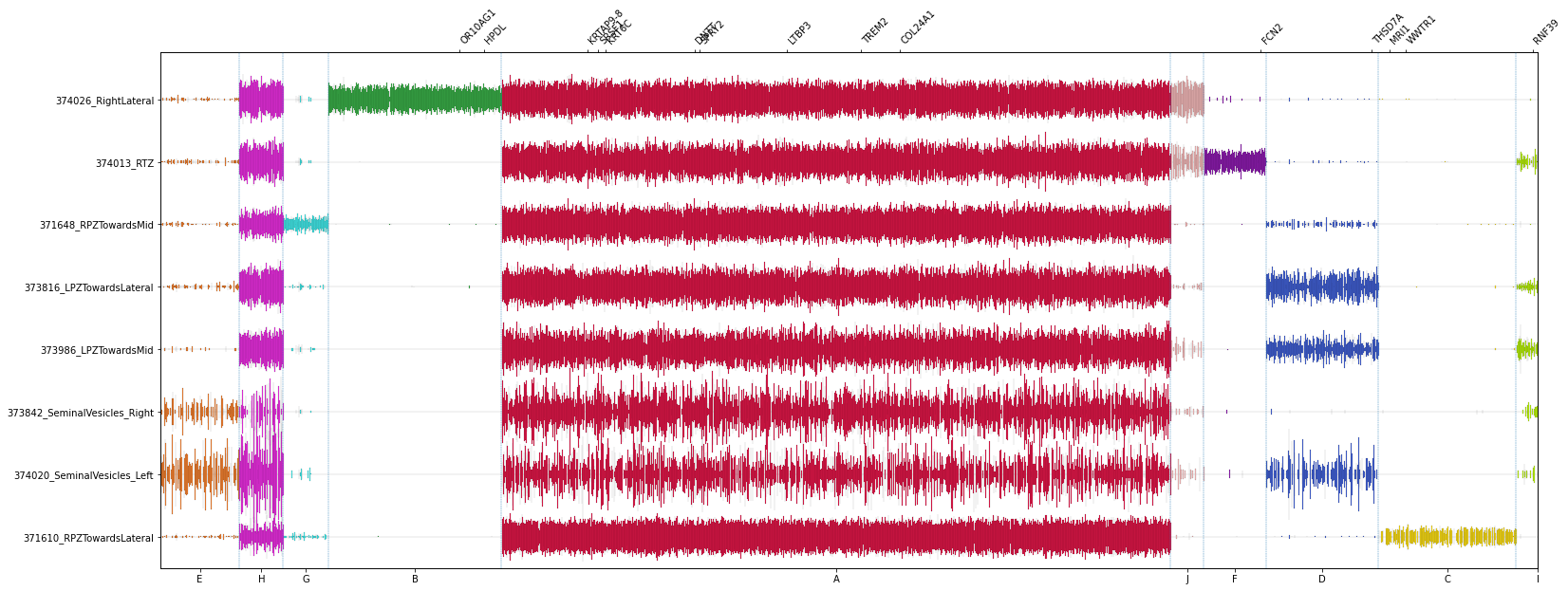
Fig. S6. SNVs from cluster I cannot be placed on the phylogenetic tree and may represent noise. Hence these SNVs were ignored.

4. **Patient #13**

Copy number profile


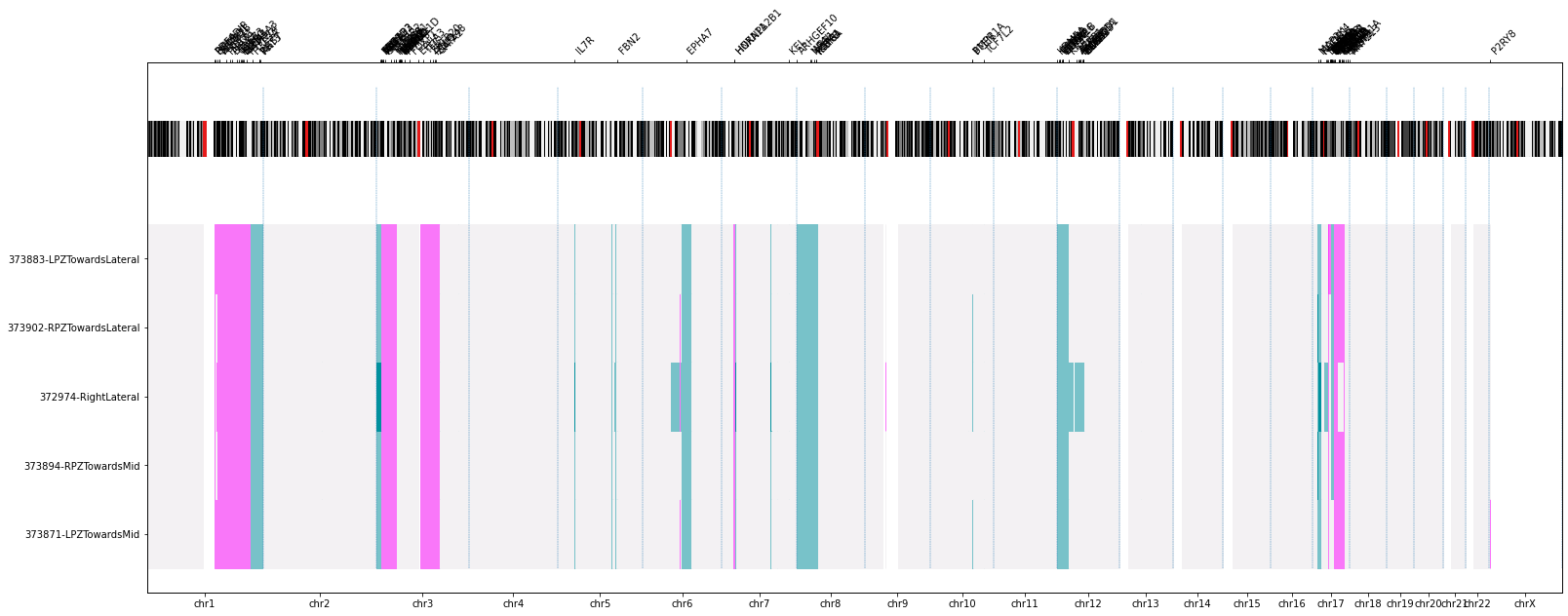
Fig. S7. 373901-Pre-prostaticFat is of lower tumour purity (0.08) and hence was assumed to have the same CNA profiles as the majority of the intra-prostatic samples; the copy number profiles for these samples are not shown.

CCF-cluster plot


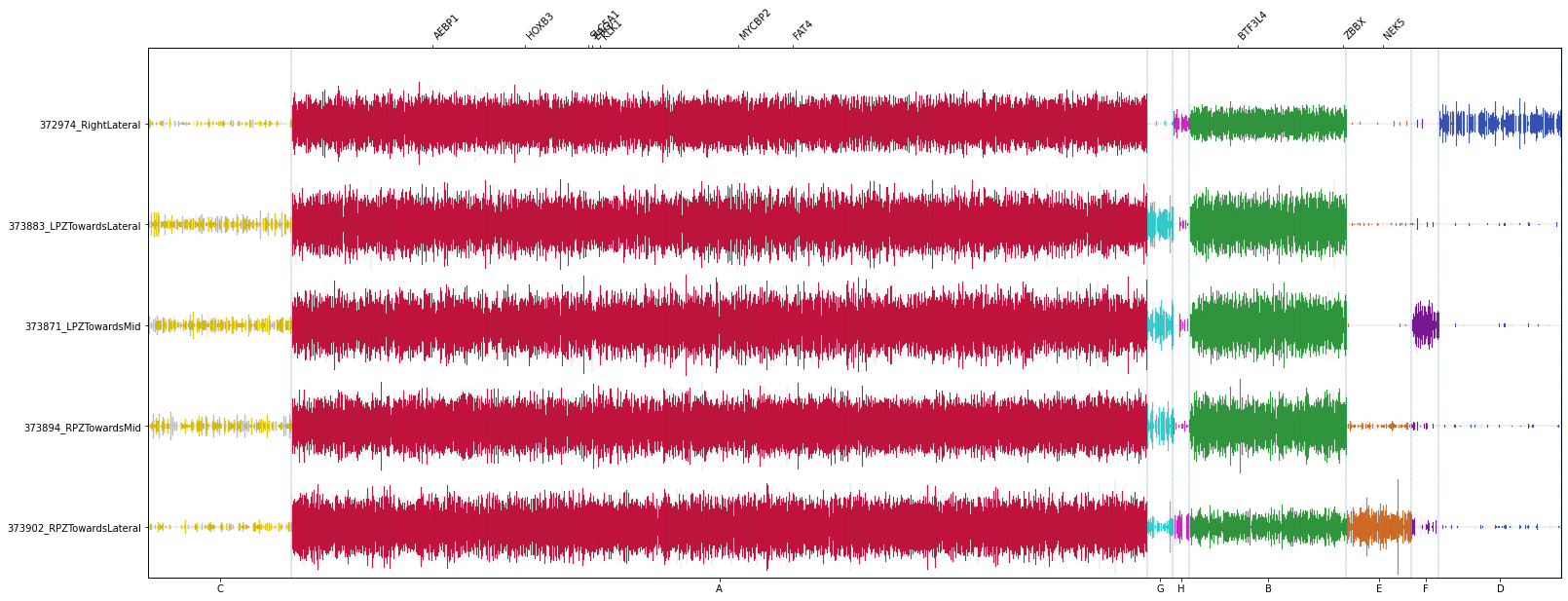
Fig. S8. SNVs from clusters G and H cannot be placed on the phylogenetic tree and may represent noise. Hence these SNVs were ignored.

5. **Patient #10**

Copy number profile


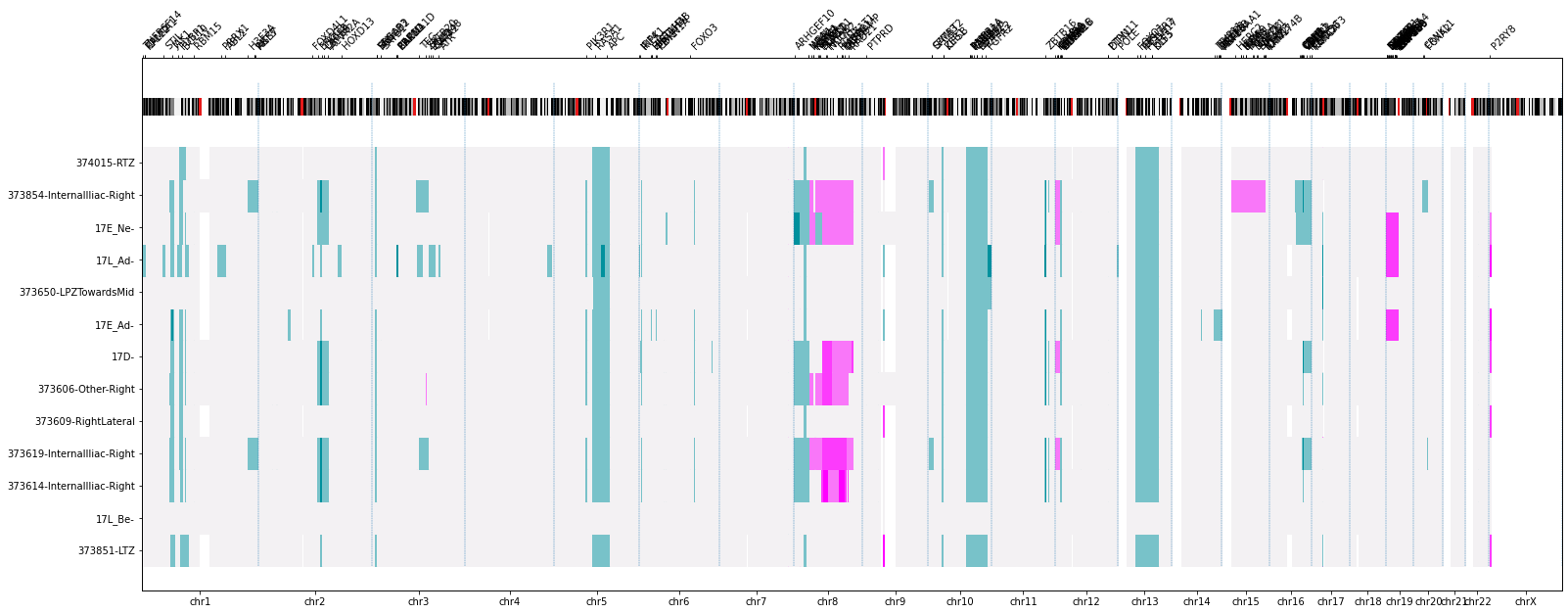
Fig. S9. Amplifications in 8p are seen in the lymph node samples and intra-prostatic neuroendocrine samples.

CCF-cluster plot


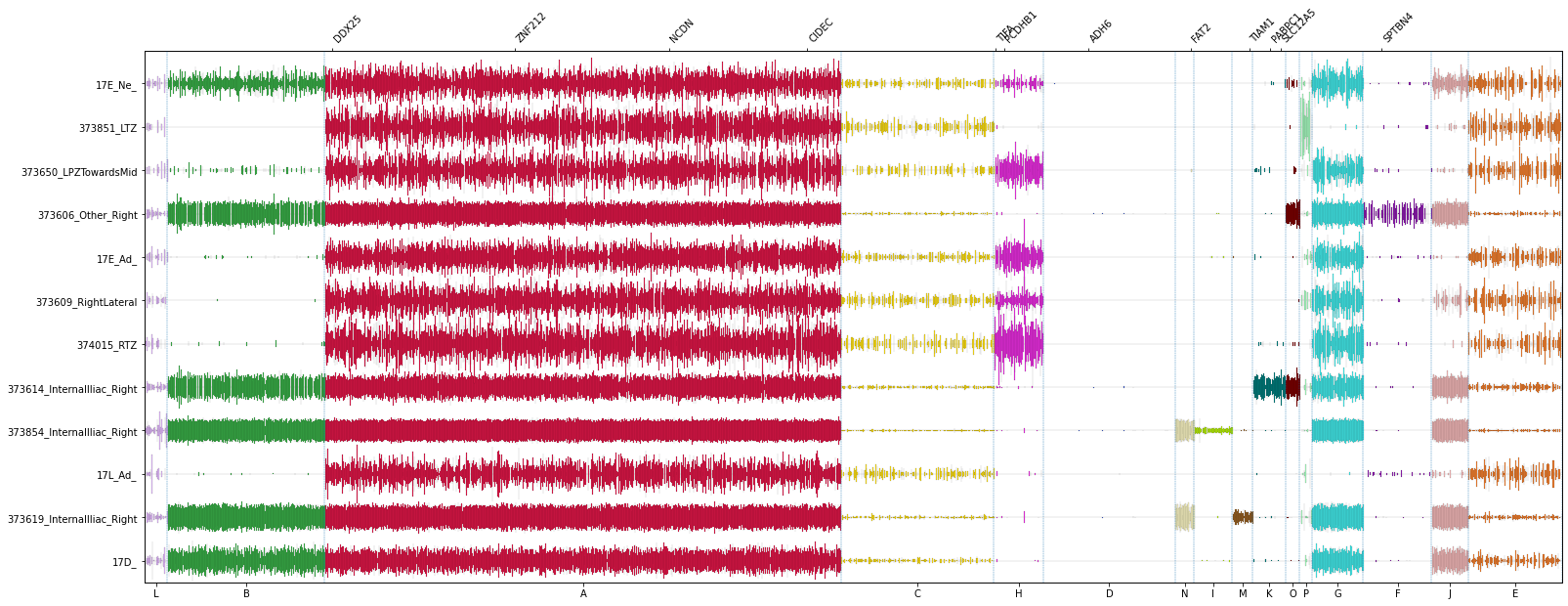
Fig. S10. SNVs from clusters L, C, P, E cannot be placed on the phylogenetic tree and may represent noise. Hence these SNVs were ignored. Cluster D is present in the Anterior sample but not in any other samples, suggesting a distinct lineage.

6. **Patient #15**

Copy number profile


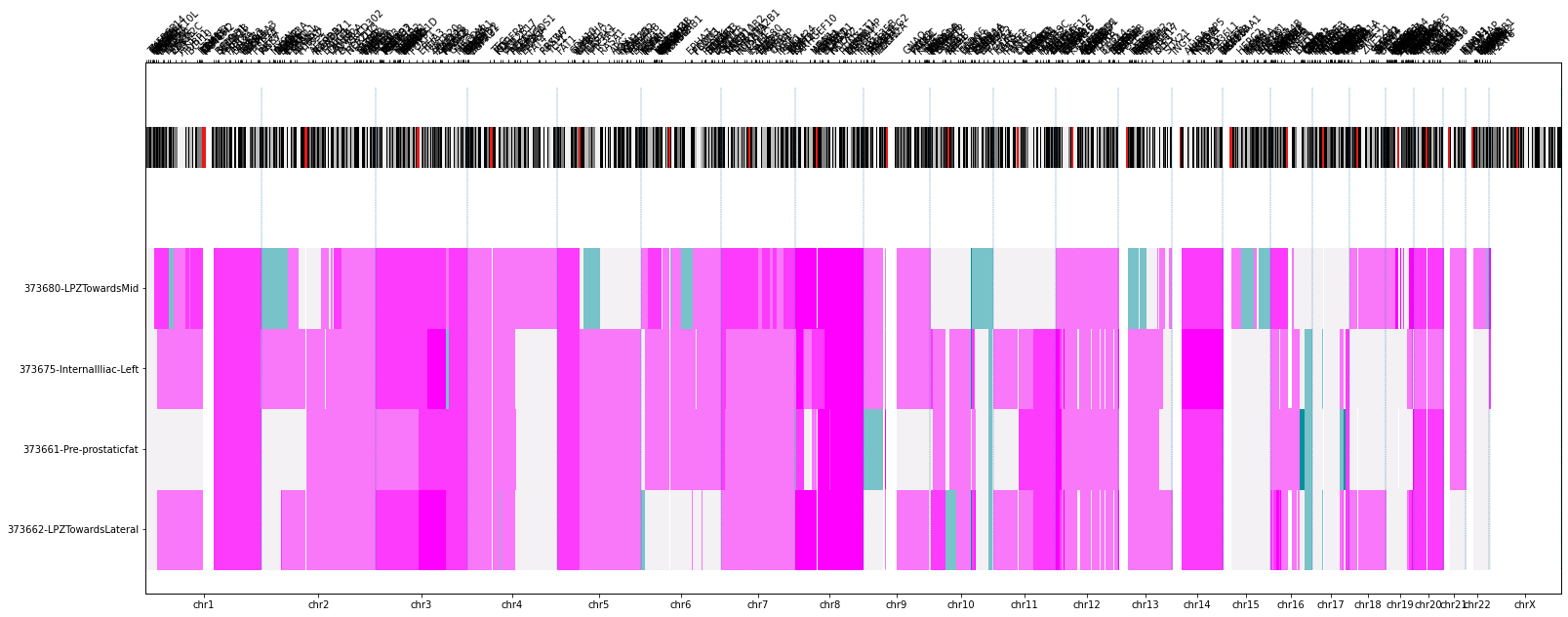
Fig. S11. Extensive copy number alteration is seen in all samples from this patient.

CCF-cluster plot


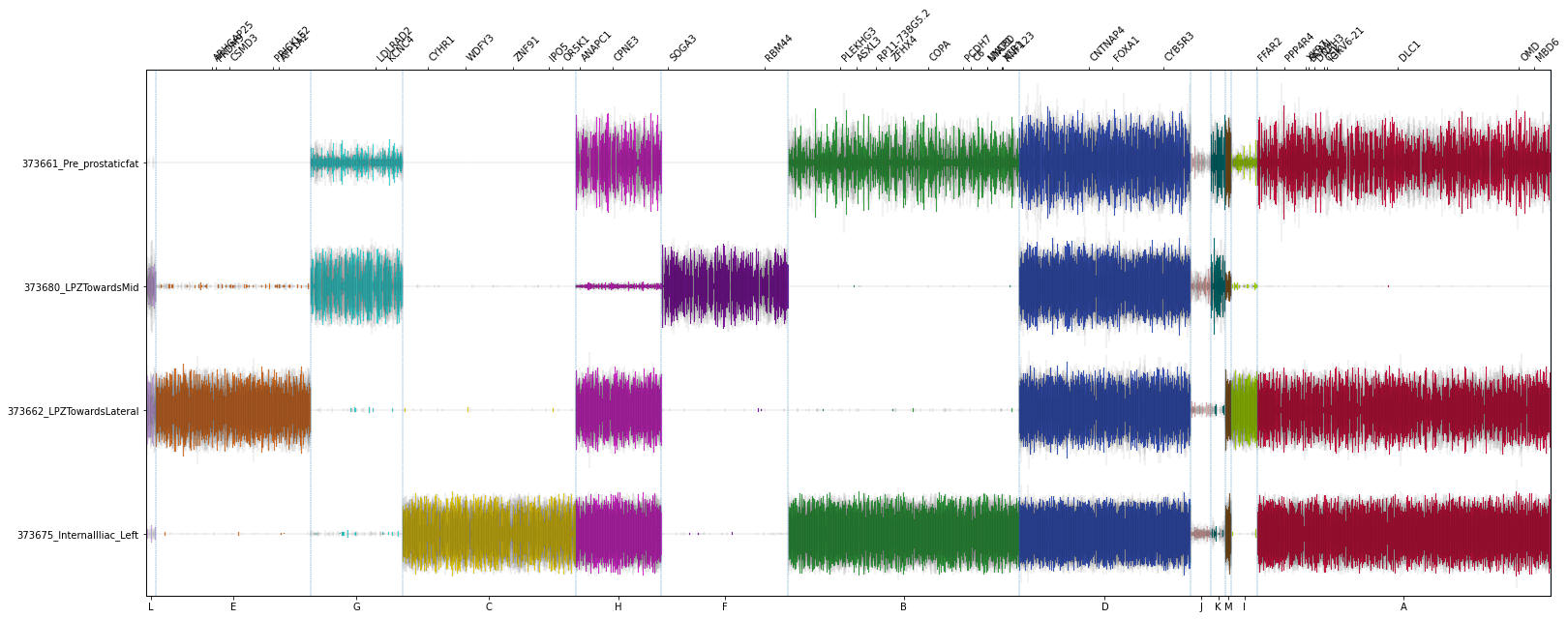
Fig. S12. SNVs from clusters L, J, K, M cannot be placed on the phylogenetic tree and may represent noise. Hence these SNVs were ignored.

7. **Knockdown of FOXP1 results in increased migration in vitro**


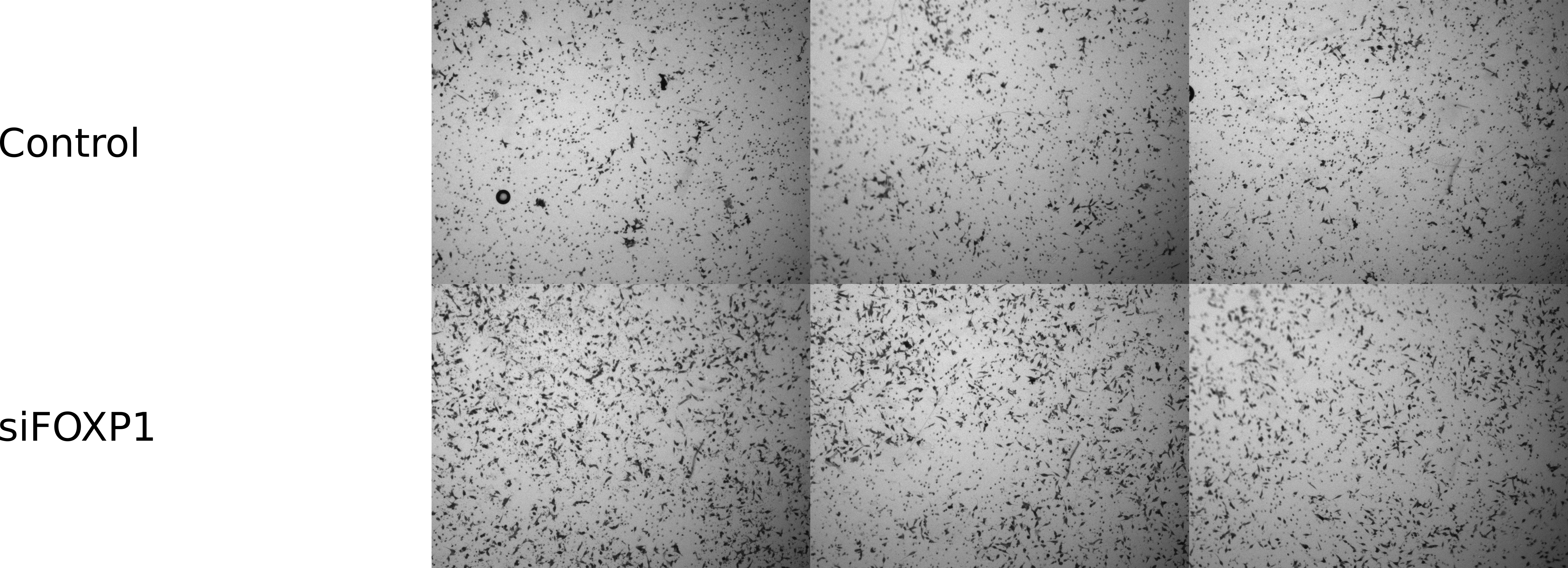
Fig. S13. LNCaP cells were transfected with control or FOXP1 siRNA and incubated in 8um transwells for 48 hours. Cells with FOXP1 knockdown have increased migration compared to control cells.

8. **Patient #15: BRCA CN status**


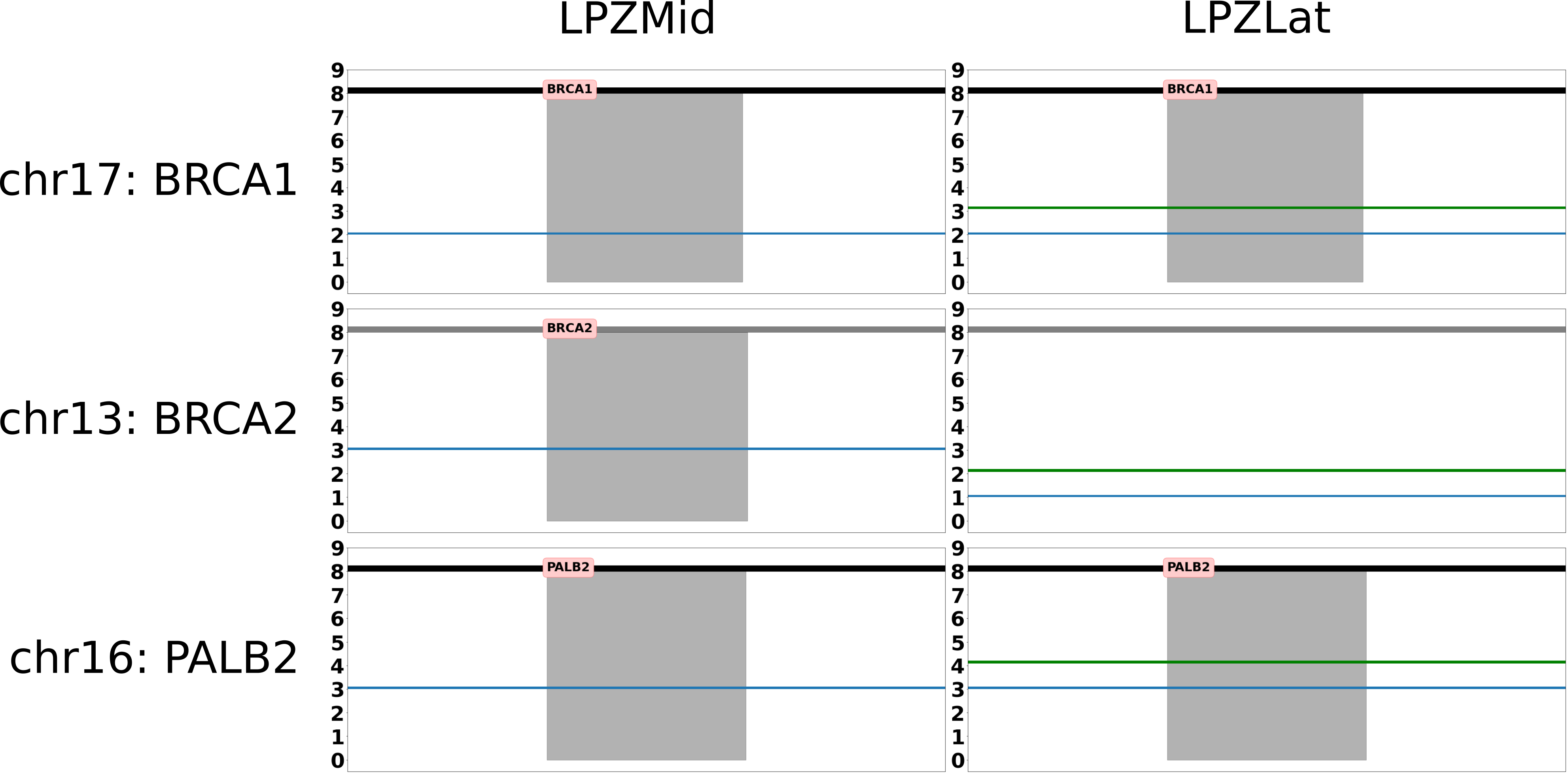


Fig. S14. Copy number status at BRCA1, BRCA2 and PALB2 loci, derived from Battenberg calls, are plotted for LPZMid and LPZLat samples. Blue line indicates major copy number and green line indicates minor copy number.

9. **Patient #15: BRCA methylation status**

BRCA1


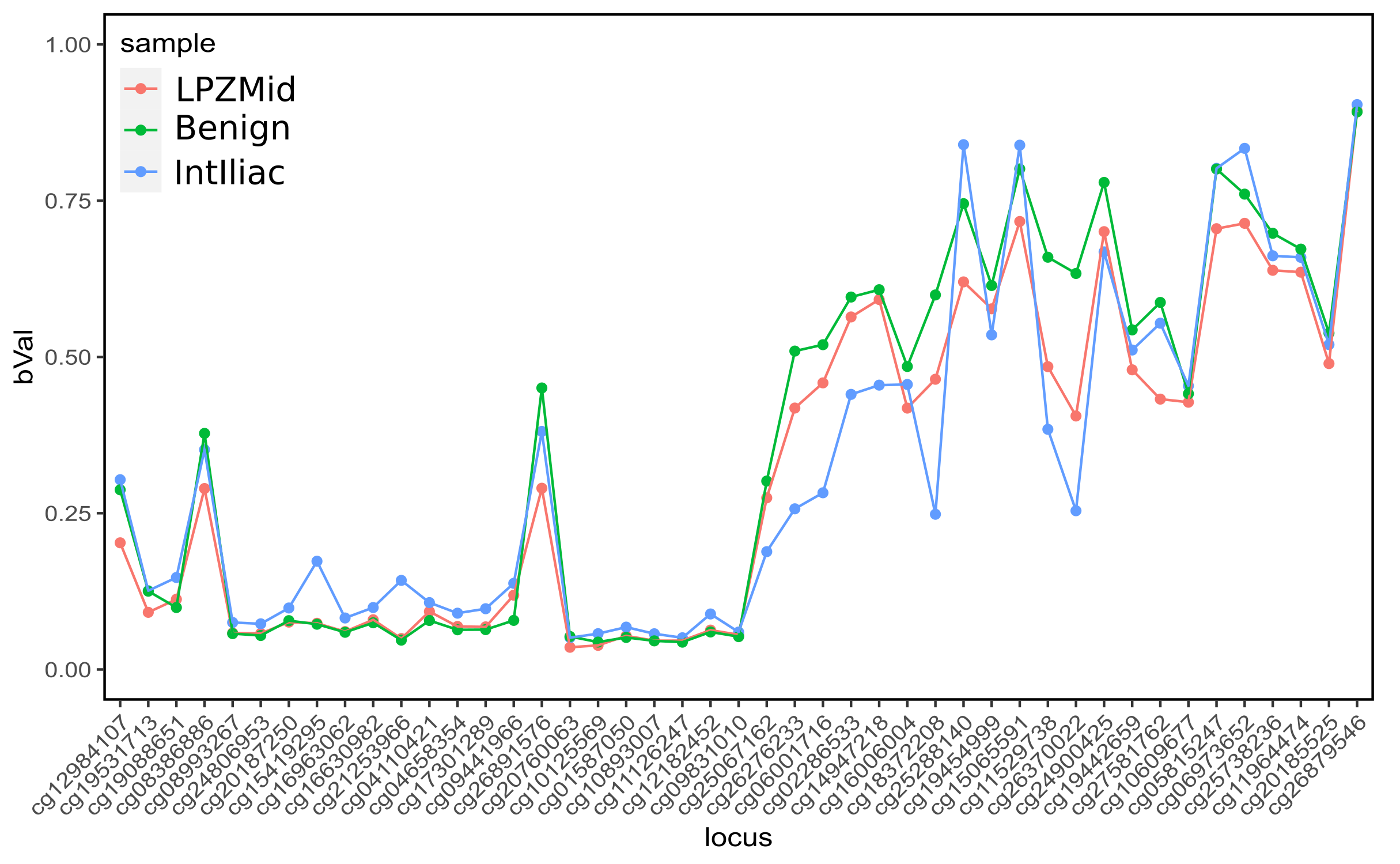
Fig. S15. Methylation levels in the BRCA1 promoter region are plotted in 3 samples from patient #15.


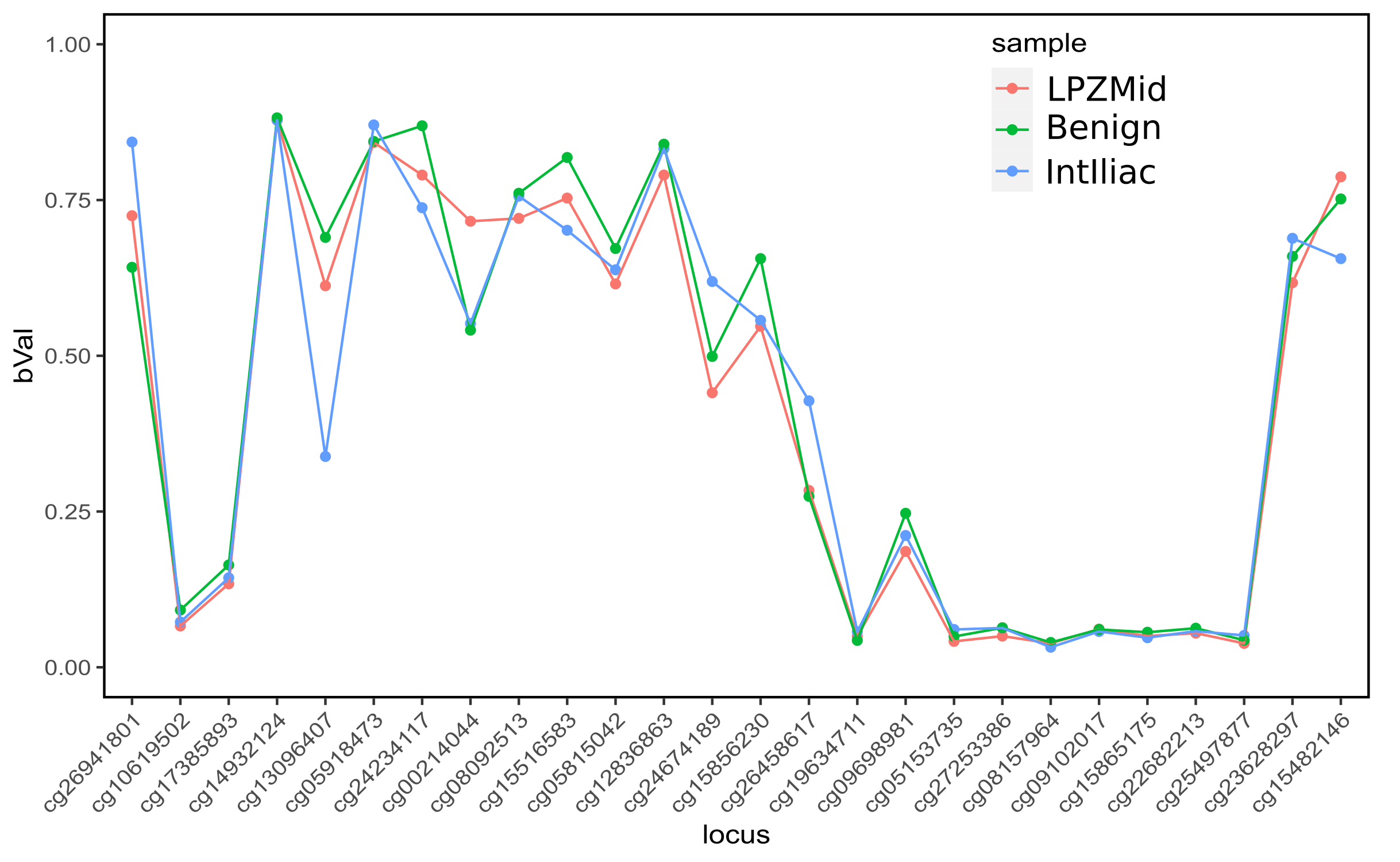
BRCA2

Fig. S16. Methylation levels in the BRCA1 promoter region are plotted in 3 samples from patient #15.

10. **Adenocarcinoma in #10**


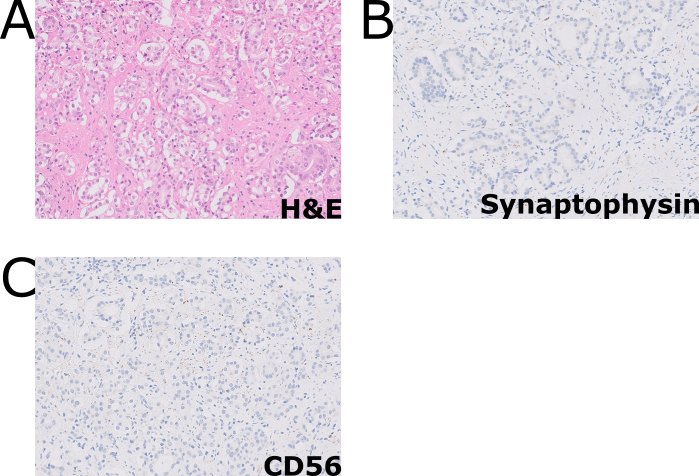


Fig. S17. The intra-prostatic tumour in patient #10 with an acinar adenocarcinoma morphology as seen by H&E (A) stained negative for the neuroendocrine markers Synaptophysin (B) and CD56 (C).


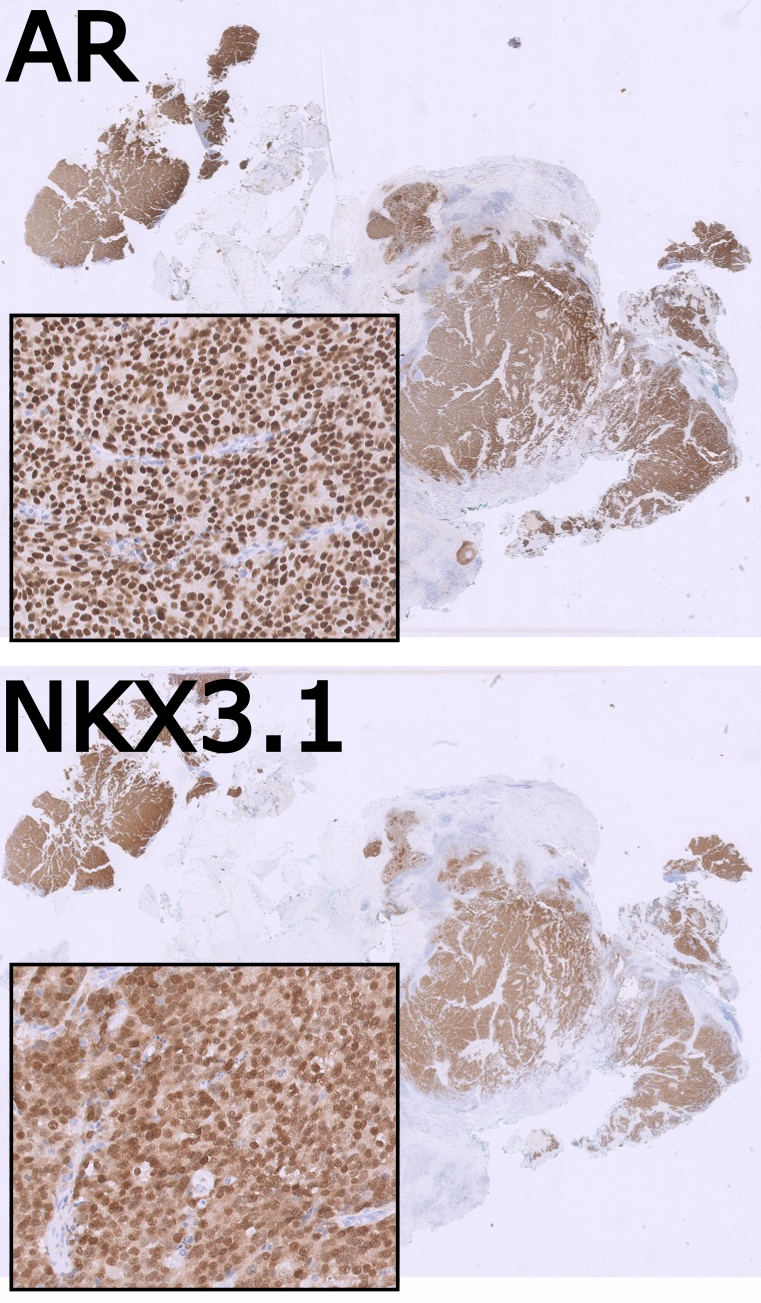


Fig. S18. The amphicrine part of the cancer in patient #10 stained positive for AR (top) and NKX3.1 (bottom).


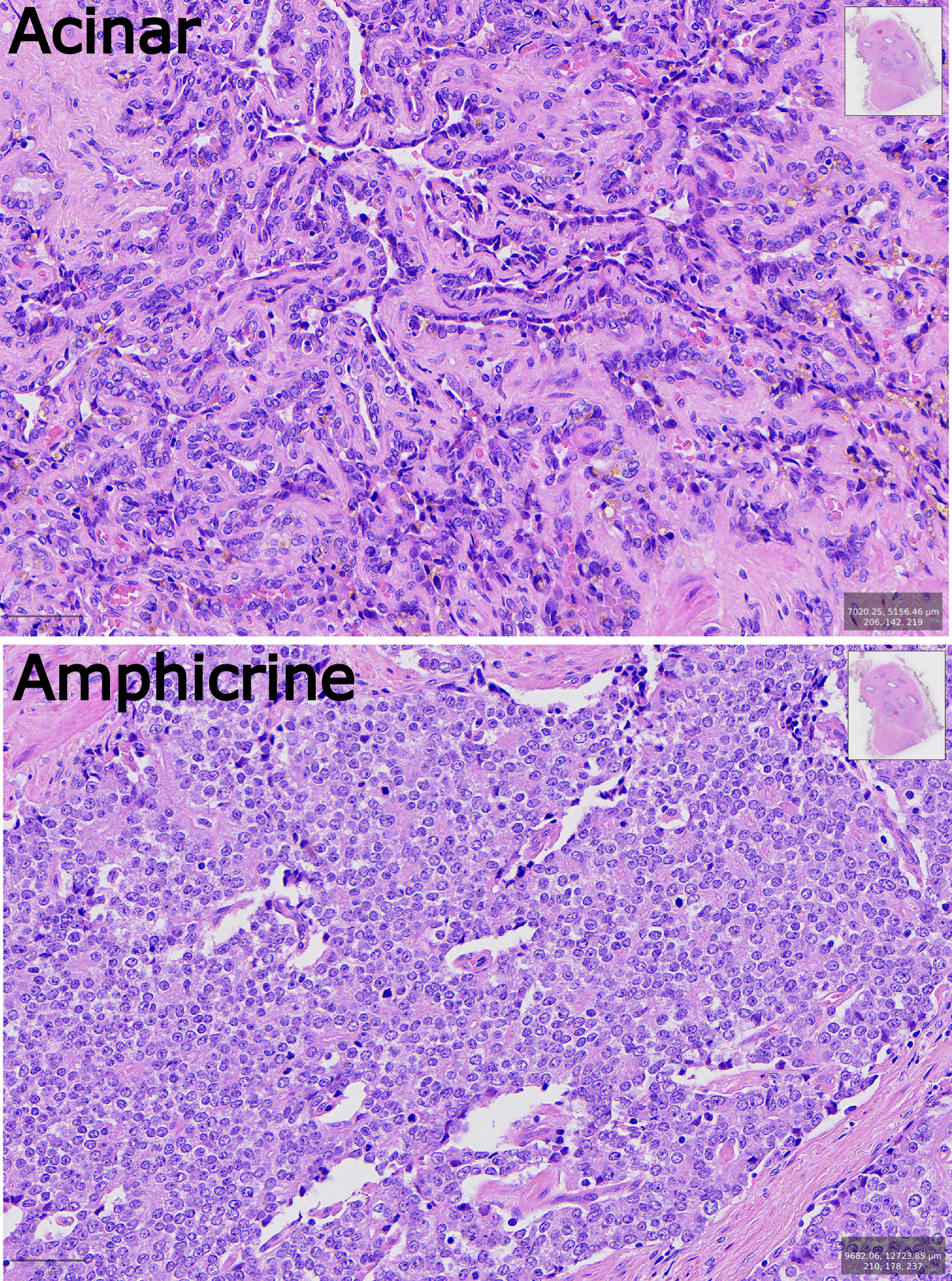


Fig. S19. The intra-prostatic tumour in patient #10 with an acinar adenocarcinoma (top) and amphicrine morphology (bottom) morphology as seen by H&E.


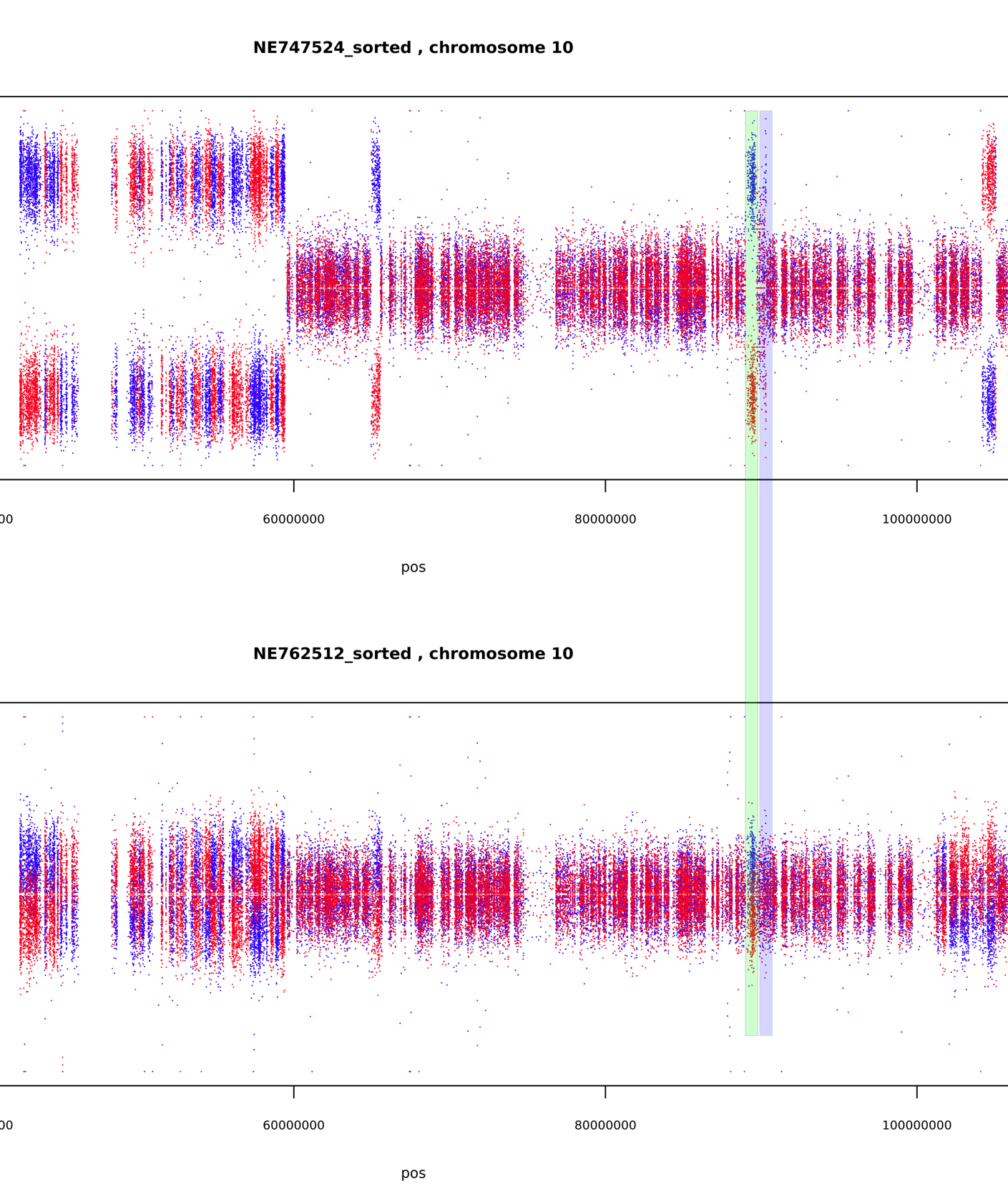


Fig. S20. B Allele Frequency plots for an intra-prostatic sample (LPZMid, top) and the seminal vesicle sample (LSV, bottom), showing the regions spanning the PTEN gene in patient 2. All samples except LSV consist of two focal lesions spanning the PTEN gene (chr10:89,623,382-89,731,687): one heterozygous loss (chr10:89,001,549-89673569; green highlight) and one homozygous deletion (chr10:89675296-90263775; blue highlight). The LSV sample was of lower purity (23%), which resulted in a lower resolution copy number profile missing both of these focal lesions. However, the B Allele Frequency plot shows evidence for at least the first (heterozygous) loss involving PTEN.

Further, 1) the PTEN loss is clonal in all intra-prostatic samples and 2) all SNVs found in LSV were a subset of those found in the intra-prostatic samples. From (1) it follows that the PTEN loss was present in clone A and all its subclones; from this and (2) it follows that invasion into LSV must have happened subsequent to PTEN loss. Hence, we infer that both focal lesions encompassing the PTEN gene must be present in LSV but were not detectable due to low purity of the sample.


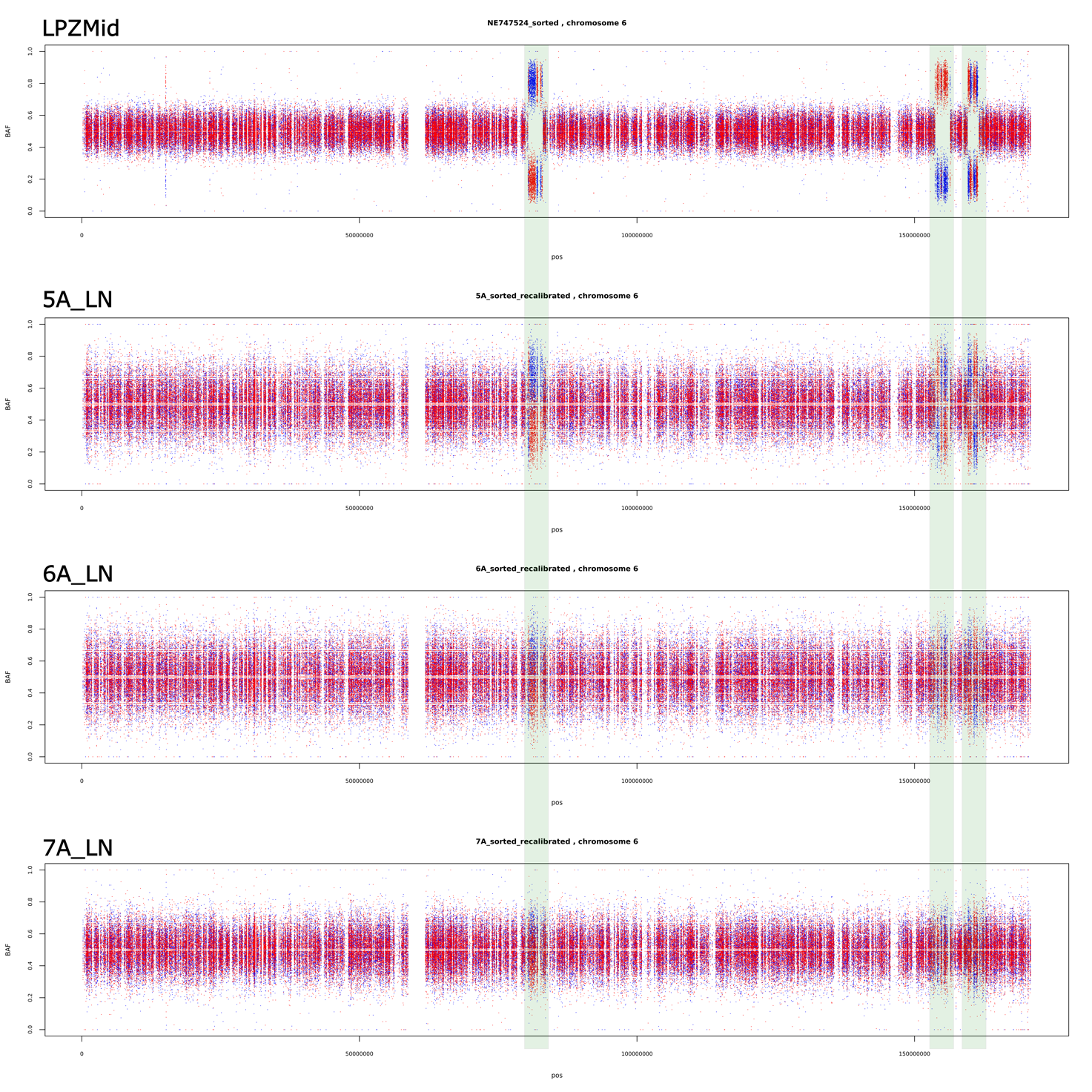


Fig. S21. B Allele Frequency plots of chromosome 6 for an intra-prostatic sample (LPZMid) and the lymph node samples (sequenced from FFPE) from patient 2, with green highlights showing regions where evidence is seen in the lymph node samples for the copy number alterations called in the intra-prostatic sample (LPZMid, top). The FFPE samples from patient 2 (5A_LN, 6A_LN, 7A_LN) were of low tumour purity as the reads from the immune cells in the LNs were in much greater numbers compared to the cancer cells. Hence, the most common CNA profile from this patient was applied to these LN samples, as stated in the methods section (‘CNA calling’). This follows the principle of parsimony (specific mutations found in the LN samples were also found in the intra-prostatic samples; based on the infinite sites model, we infer that the LN samples share a phylogenetic history with the intra-prostatic samples). As the copy number alterations are present clonally in the ancestral clone, we infer that at least the same CNAs must be present in the LN samples as well. Furthermore, we show the B Allele Frequency plots from the LN samples below and compare them to that from an intra-prostatic sample to show that there is evidence supporting these copy number changes in the LN samples, even though they were not called by Battenberg due to low purity.
